# Efficacy and safety of Chinese patent medicine compound preparation combined with routine treatment in vitiligo: A Bayesian network meta-analysis

**DOI:** 10.1097/MD.0000000000035327

**Published:** 2023-10-13

**Authors:** Jianfeng Wang, Dingding Wang, Guomin Si

**Affiliations:** a Shandong University of Traditional Chinese Medicine, Jinan, Shandong, China; b Jining Hospital of Traditional Chinese Medicine, Jining, Shandong, China; c Department of Traditional Chinese Medicine, Shandong Provincial Hospital, Jinan, Shandong, China.

**Keywords:** Bayesian network meta-analysis, Chinese patent medicine, efficacy, safety, vitiligo

## Abstract

**Background and purpose:**

Treating vitiligo in clinical practice is challenging. Furthermore, oral drugs used in Western medicine have considerable side effects and are unsuitable for long-term treatment. In contrast, Chinese patent medicines (CPMs) are more suitable for long-term oral vitiligo treatment, but medical evidence of their efficacy and safety is lacking. Therefore, in this study, the efficacy and safety of CPMs were evaluated and ranked using a Bayesian network meta-analysis.

**Methods:**

Seven Chinese and English databases were searched for all relevant articles published up to February 2023. The Bayesian network meta-analysis method was used to analyze the extracted data to evaluate efficacy and safety.

**Results:**

Six common CPMs for treating vitiligo were selected in our study, and 48 targeted articles and 4446 patients were included. This study showed that Qubai Babuqi tablets (QT) were the most effective for short-term treatment of vitiligo, and that vitiligo capsules or pills (VCP) were the most effective for long-term treatment, together with compound Quchong Banjiuju pills (QP). In terms of surface area under the cumulative ranking curve (SUCRA) values, the order of efficacy of each treatment was as follows: QT (92.18%) > Taohong Qingxue pills (TP) (63.81%) > VCP (55.53%) > QP (50.72%) > Bailing tablets or capsules (BTC) (49.01%) > Baishi pills (BP) (35.69%)>routine therapy (RT) (3.1%) in terms of total effective rate and QT (92.05%) > VCP (71.50%) > QP (66.60%) > TP (42.95%) > BTC (39.66%) > BP (36.60%)>RT (0.6%) in terms of improvement rate. In addition, the safety of the 6 CPMs did not significantly differ in terms of adverse effects. The SUCRA values indicated that QT performed slightly worse than other drugs.

**Discussion:**

In treating vitiligo, QT is most effective but only suitable for short-term administration owing to its poor safety. VCP and QP could be used as first-choice long-term medications. TP may positively affect repigmentation in patients with limited lesion areas.

## 1. Introduction

Vitiligo is an acquired depigmentation disease of the skin or mucosa, which can occur in any part of the body, particularly exposed skin and areas susceptible to friction.^[1]^ The typical clinical manifestation of vitiligo is a sharply defined, non-scaly opalescent macula with occasional normal skin islands.^[2]^ Dermoscopy can be used to estimate disease activity and prognosis, helping to formulate personalized treatment schemes.^[3]^ Vitiligo is divided into localized, generalized, and universal types according to the distribution range of skin lesions. In addition, the distribution of vitiligo is classified as segmental or non-segmental. In addition, there are subtypes of vitiligo, such as mucous (involving the mucous membrane) and facial or acromelic (involving the face or the distal end of limbs). Different subtypes of vitiligo can coexist in the same patient.^[4,5]^ Vitiligo patients often have a series of psychological symptoms or disorders caused by skin depigmentation and the features of the exposed sites, which seriously affect their daily and social lives.^[6]^ In China, the latest multicenter epidemiological study showed that the prevalence of vitiligo was 0.56%, representing an increase from previous studies.^[7]^ Localized vitiligo was the most common form (36.1%), males were affected more than females, and prevalence increased with age. Approximately 9.8% of patients had a positive family history.^[7]^ Recent epidemiological studies on vitiligo in the United States estimate the overall morbidity in adults from the United States at 0.76% to 1.11%. In addition, it has been suggested that approximately 40% of adults in the United States may remain undiagnosed.^[8]^

Currently, the main treatment measures for vitiligo include oral or topical corticosteroids, topical calcineurin inhibitors, phototherapy or photochemotherapy, topical photosensitizers (such as psoralen and nitrogen tincture), and autoepidermal graft techniques.^[5]^ Following the discovery of the Janus kinase (JAK)/signal transducer and activator of transcription (STAT) pathways as a significant factor in the pathogenesis of vitiligo, treatment options have expanded to include Lucotinib, the first cream of its type approved by the FDA to induce repigmentation in vitiligo.^[9]^ Traditional Chinese medicine is also widely applied in treating vitiligo. According to the work of an ancient doctor named Chao Yuanfang in the Sui Dynasty, vitiligo was termed “Baidian,” which is its earliest recorded description in China. Many famous Chinese medicine practitioners of successive dynasties adopted methods promoting blood circulation, removing wind pathogens, and reinforcing kidneys to manage vitiligo.^[10]^ Through long-term accumulation of experience in clinical and modern drug preparation, a series of Chinese patent medicine (CPM) compound preparations for vitiligo treatment have been developed. However, the therapeutic effects and safety of proprietary Chinese medicines remain unclear. Our study aimed to investigate and rank the efficacy and safety of common CPMs using a Bayesian network meta-analysis to provide effective evidence for applying CPMs in the treatment of vitiligo. The authors preliminarily identified 6 commonly used CPMs from the literature as research subjects for analysis, namely vitiligo capsules or pills (VCP), Bailing tablets or capsules (BTC), Qubai Babuqi tablets (QT), Baishi pills (BP), Taohong Qingxue pills (TP), and compound Quchong Banjiuju pills (QP). Network meta-analysis (NMA) considers direct and indirect comparisons and compensates for the disadvantages of simple direct comparisons in ordinary meta-analyses.

## 2. Methods

The meta-analysis of randomized controlled trials (RCTs) in our study was conducted according to the PRISMA statements registered on the PROSPERO website registration platform (Registration Number: CRD42022375977). However, the protocol was not prepared.

### 2.1. Inclusion criteria

The following inclusion criteria applied. Study type: all included studies were RCTs. Patients: the selected patients had a diagnosis of vitiligo based on diagnostic standards according to international and Chinese expert consensus and authoritative dermatology books.^[11–19]^ All types of vitiligo were included, and the age and sex of the patients were not restricted. Interventions: the treatment measures of each intervention arm included one of the target CPM compound preparations: VCP, BTC, QT, BP, TP, and QP. The other arm used only conventional treatments for vitiligo: corticosteroids, calcineurin inhibitors, immunomodulators, phototherapy, photosensitizers, and the autoepidermal graft technique. RCTs that reported trials comparing target CPMs were included. Outcomes: at least one of the following results was documented in the studies: efficacy, which included total effective rate and good improvement rate; adverse reactions; skin lesion pigment score; area of the lesion; and quality-of-life score according to the Dermatosis Life Quality Index. The skin lesion pigment score was evaluated according to the skin color of the patient. The scores ranged from 0 to 3, with zero indicating that the lesions were pure or milky white with no pigment. A score of 1 indicated that the skin was lightly white with a small amount of pigmentation. A score of 2 indicated that the skin color was light brown with a large amount of pigmentation. A score of 3 indicated that the skin had returned to normal.^[5]^ The efficacy criterion was as follows: healing, when white spots had disappeared completely and the normal skin accounted for 100% of the total area of skin lesions. This indicated a drug preference when the percentage ranged from 50% to 99% of the total skin lesion area. If the proportion was <50% or more than 10%, the efficacy was determined as improved.^[5]^

### 2.2. Exclusion criteria

The following exclusion criteria applies: studies not published as RCTs, no definite diagnostic and efficacy criteria, unqualified interventions, basic research, reviews, or meta-analyses, duplicate publications, and missing key data.

### 2.3. Search strategy

Seven Chinese and English databases were searched for relevant articles dated up to February 2023: PubMed, Cochrane Library, Embase, Web of Science, China National Knowledge Infrastructure (CNKI), Wanfang Data, and China Science and Technology Journal Database (VIP). In order to prevent the omission of eligible studies, we have standardized the retrieval strategy according to the participants, intervention, comparison, outcomes, and study design (PICOS) principles. Our retrieval strategy is mainly limited to participants, intervention measures and research types. In addition, RCTs that reported on the efficacy and safety of the target drugs for treating vitiligo were selected. The search terms and synonyms used were “vitiligo,” “leukoderma,” “Chinese patent medicine,” “proprietary Chinese medicine,” “Chinese patent drug,” “traditional Chinese medicine,” “Chinese medicine,” “Vitiligo capsules,” “Vitiligo pills,” “Baidianfeng capsules,” “Baidianfeng pills,” “ Anti-vitiligo capsules,” “Bailing tablets,” “Bailing capsules,” “Qubai Babuqi tablets,” “Qubaibabuqi tablets,” “Baishi pills,” “White Corrosion pills,” “Taohong Qingxue pills,” “Taohong Qingxue tablets,” “compound Quchongbanjiuju pills,” “Compound vernonia anthelmintica pills,” and “Bairesi pills.” The retrieval strategy for PubMed is shown in Table 1.

**Table 1:** Search strategy (Pubmed). The retrieval strategy of PubMed is set out in this diagram as an example.

#1 vitiligo [MeSH Terms]
#2 vitiligo [Title/Abstract]
#3 leukoderma [Title/Abstract]
#4 #1 OR #2 OR #3
#5 “Chinese patent medicine” [Title/Abstract]
#6 “Chinese patent drug” [Title/Abstract]
#7 “proprietary Chinese medicine” [Title/Abstract]
#8 “medicine, Chinese traditional” [MeSH Terms]
#9 “traditional Chinese medicine” [Title/Abstract]
#10 “Chinese medicine” [Title/Abstract]
#11 “Vitiligo capsules” [Title/Abstract]
#12 “Baidianfeng capsules” [Title/Abstract]
#13 “Vitiligo pills” [Title/Abstract]
#14 “Baidianfeng pills” [Title/Abstract]
#15 “Anti-vitiligo capsules” [Title/Abstract]
#16 “Bailing tablets” [Title/Abstract]
#17 “Bailing capsules” [Title/Abstract]
#18 “Qubai Babuqi tablets” [Title/Abstract]
#19 “Qubaibabuqi tablets” [Title/Abstract]
#20 “Baishi pills” [Title/Abstract]
#21 “White Corrosion pills” [Title/Abstract]
#22 “Taohong Qingxue pills” [Title/Abstract]
#23 “Taohong Qingxue tablets” [Title/Abstract]
#24 “compound Quchongbanjiuju pills” [Title/Abstract]
#25 “Compound vernonia anthelmintica pills” [Title/Abstract]
#26 “Bairesi pills” [Title/Abstract]
#27 #5 OR #6 OR #7 OR #8 OR #9 OR #10 OR #11 OR #12 OR #13 OR #14 OR #15 OR #16 OR #17 OR #18 OR #19 OR #20 OR #21 OR #22 OR #23 OR #24 OR #25 OR #26
#28 “randomize controlled trial” [Publication Type]
#29 “clinical trial” [Publication Type]
#30 randomize [All Fields]
#31 trial [All Fields]
#32 study [All Fields]
#33 #27 OR #28 OR #29 OR #30 OR #31
#34 English [Language]
#35 #4 AND #27 AND #33 AND #34

### 2.4. Literature screening and data extraction

Two reviewers retrieved literature in strict accordance with the inclusion and exclusion criteria. After screening for eligibility, 2 different reviewers recorded the data of the included studies as follows: title, first author, publication year, sample capacity of each arm, sex, age, disease course, interventions, intervention course, and outcomes. Finally, another reviewer verified the data selection. If puzzlement or bifurcation occurred, or any of the above data were dropped or recorded incompletely, we contacted the original author to notarize and complete the data.

### 2.5. Risk of bias assessment

The bias risk of our study was estimated by 2 non-interfering reviewers using the Cochrane risk of bias tool.^[20]^ This tool was used to appraise the quality of every selected article, including random sequence generation, allocation concealment, whether studies were blinded or not, incomplete data, selective reporting, and other biases. Each study was sorted into high, low, or unclear risk categories for inclusion. If one of the entries was categorized as high-risk, the overall quality was also estimated as high-risk.^[21]^

### 2.6. Statistical analysis

Considering that the main indicators, that is, the total effective rate, good improvement rate, and adverse effects, are binary variables, the odds risk (OR) and 95% confidence interval (CI) were used in our study as the effect size. The remaining outcomes were continuous variables. Therefore, this study used the mean difference (MD) and 95% CI as the effect size. Standardized mean differences (SMDs) were adopted when the mean values differed significantly between the studies.^[22]^ The WINBUGS software (Microsoft, 14.0) was used to conduct a Bayesian network meta-analysis. Four Markov chains were used for simulation, and the number of iterations was set to 50,000 (the first 20,000 were used for annealing to eliminate the influence of the initial value, and the last 30,000 were used for sampling).^[22]^ We set a minimal baseline prior distribution and a deviation parameter to evaluate heterogeneity between studies. The posterior statistics of each group of data collected are calculated by the Markov Chain Monte Carlo method, and the sum of deviation parameters was compared with the total number of research arms. If both values were approximate, this showed that the model used in statistics was a good fit and the results were reasonable. STATA (StataCorp LLC, 14.0) software was adopted for the generation of network diagrams, ranking plots, and league tables. Consistency was tested using the Chi-square test. When I^2^ was not significant (I^2^ < 50%, *P* > .05), the direct comparison was consistent with the result of the indirect comparison.^[23]^ Next, inconsistency and heterogeneity tests were performed using inconsistency factors (IFs) in STATA 14.0. The efficacy of each CPM was ranked according to surface area under the cumulative ranking curve (SUCRA) values. As an increase in the total effective rate and good improvement rate was a beneficial outcome, high SUCRA values correlated with good efficacy. A funnel plot was used to determine publication bias.

## 3. Results

### 3.1. Study selection and characteristics

**Figure 1.**
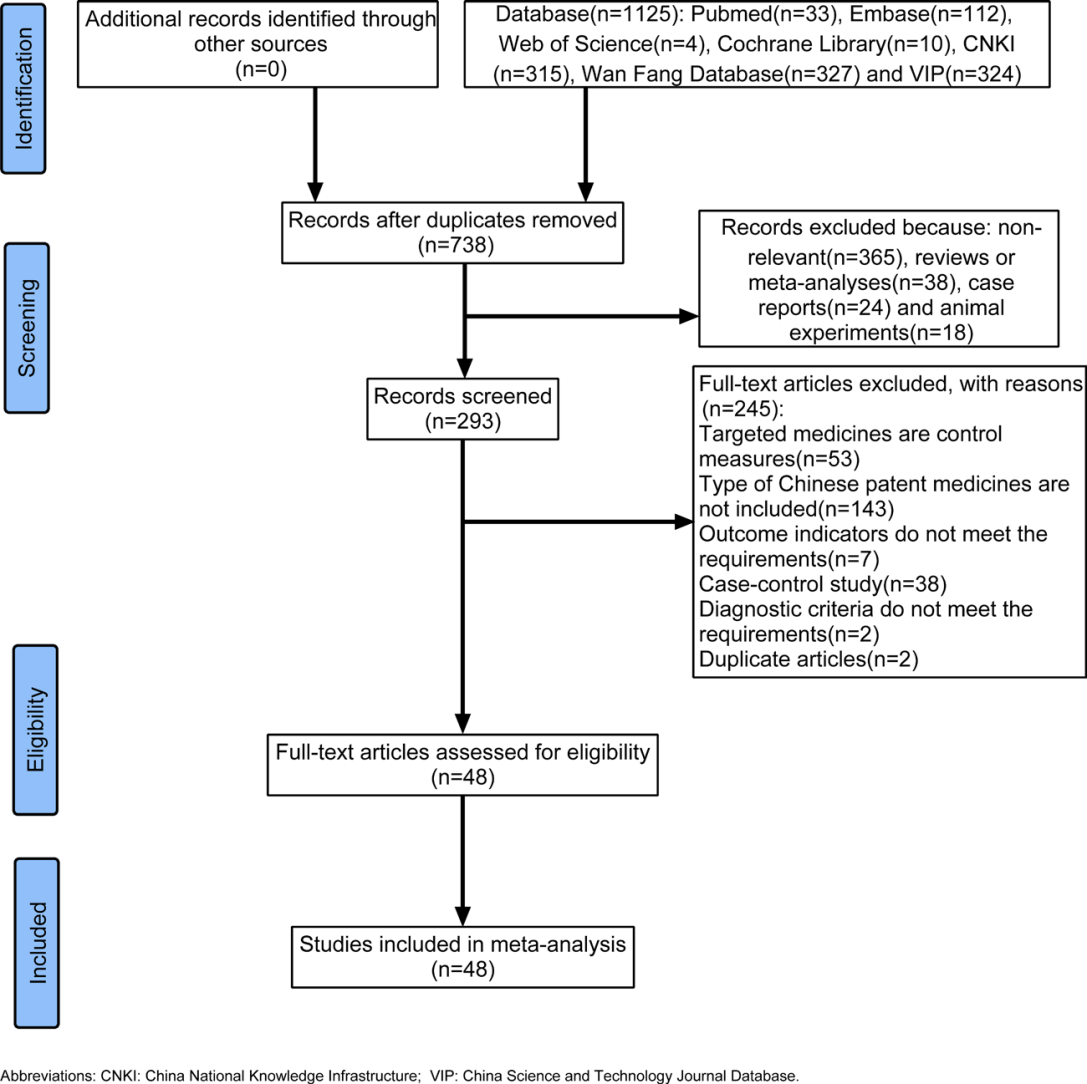
Flow chart of literature screening.

A total of 1125 articles were retrieved from PubMed (n = 33), Embase (n = 112), the Cochrane Library (n = 10), CNKI (n = 315), Wanfang (n = 327), and VIP (n = 324). This decreased to 738 after duplicate articles were removed. We screened the titles and abstracts of these articles. Some were excluded as they were narrative reviews and meta-analyses (n = 38), case reports (n = 24), animal experiments (n = 18), or had no relation to our research (n = 365). After the screening, the full text was downloaded and read. In total, 143 articles were excluded for the following reasons: irrelevant CPMs (n = 143), unqualified control measures (n = 53), unmatched outcome indicators (n = 7), non-RCTs (n = 38), unclear diagnostic criteria (n = 2), and suspected duplications (n = 2). Finally, 48 articles^[24–71]^ were included in this study. In our research, 4446 patients were included, with 1193 patients in the experimental group and 2153 patients in the control group. The literature screening process is shown in Figure 1, and the basic information of the included studies is shown in Table 2.

**Table 2:** Characteristics of included studies.

First author/yr	Sample size		Sex (M/F)		Age (yr)		Course of disease (yr)		Treatment		Course (mo)	Measuring indicators
	s	CG	EG	CG	EG	CG	EG	CG	EG	CG		
Shao W.J. 2023^[24]^	42	42	22/20	20/22	53.59 ± 5.28	53.64 ± 5.33	-		Es	G	3	②③④⑤⑥
Yu Q. 2022^[25]^	39	39	23/16	22/17	33.17 ± 7.20	33.20 ± 7.09	12.37 ± 3.21	12.43 ± 3.24	A	G	3	①②③④
Teng J.N. 2022^[26]^	24	24	7/17	9/15	32.25 ± 14.04	25.21 ± 15.42	2.00 ± 4.07	1.50 ± 0.74	F	G	12	①②③
Deng J.L. 2022^[27]^	31	31	14/17	15/16	35.08 ± 5.32	35.01 ± 5.24	6.91 ± 1.10	6.85 ± 1.02	E	G	3	①②③④⑤
Zhao J 2021^[28]^	10	10	7/3	6/4	42.17 ± 1.40	42.20 ± 1.40	6.04 ± 1.00	6.04 ± 1.10	C	G	12	①②③
Zhang Y. 2021^[29]^	25	25	13/12	12/13	30.32 ± 5.53	29.35 ± 5.62	3.67 ± 1.19	3.24 ± 1.23	A	G	1	①②
Zhang L.J. 2021^[30]^	108	105	56/52	54/51	32.60 ± 9.60	33.20 ± 10.10	7.10 ± 4.90	6.90 ± 3.80	E	G	3	①②
Zhang J.S. 2021^[31]^	78	78	40/38	43/35	37.20 ± 2.42	37.10 ± 2.55	-		C	G	2	②
Xu Y.Q. 2021^[32]^	31	31	17/14	18/13	36.50 ± 11.50	36.00 ± 11.75	-		A	G	1	②
Shi Z.W. 2021^[33]^	34	34	15/19	16/18	28.12 ± 2.43	28.31 ± 2.14	13.12 ± 2.11	14.21 ± 2.33	C	G	1.5	①②
Ma L.H. 2021^[34]^	50	50	23/37	21/29	31.45 ± 1.56	31.12 ± 1.34	-		A	G	2	②③
Kang L. 2020^[35]^	50	50	26/24	25/25	29.54 ± 11.29	28.43 ± 10.38	6.69 ± 2.06	6.86 ± 2.12	C	G	3	②③
Ha D. 2020^[36]^	45	45	49/41		35.16 ± 2.03		3.17 ± 0.13		A	G	1	②
Yin H.Y. 2019^[37]^	50	50	24/26	27/23	35.60 ± 0.80	34.8 ± 1.10	4.03 ± 0.90	4.30 ± 0.80	A	G	2	②③
Yi Q.L. 2018^[38]^	32	32	41/54		28.00 ± 6.67		1.60 ± 0.96		B	G	4	②③
Xia Y. 2018^[39]^	60	60	58/62		-		-		C	G	4	①②③
Liu J.J. 2018^[40]^	60	60	98/82		39.54 ± 4.55		12.78 ± 3.24		C	G	3	①②
Zheng W.L. 2017^[41]^	50	50	13/37	10/40	31.22 ± 10.87	32.46 ± 10.27	2.50 ± 1.27	2.46 ± 1.24	C	G	3	①②③⑥
Wang X.H. 2017^[42]^	15	15	9/6	8/7	35.70 ± 2.44	36.50 ± 3.55	-		C	A	2	①②
Wang J. 2017^[43]^	40	40	22/18	26/14	45.70 ± 10.10	46.50 ± 9.30	7.10 ± 2.40	6.80 ± 1.70	A	G	3	①②③
Wang F. 2017^[44]^	35	35	13/22	14/21	34.52 ± 13.26	34.52 ± 13.26	2.23 ± 1.75	2.23 ± 1.75	B	G	6	①②③
Lu Z.Q. 2017^[45]^	49	49	32/17	30/19	24.60 ± 4.50	25.40 ± 4.10	-		A	G	4	①②⑤
Xie S.H. 2017^[46]^	34	35	18/16	20/15	22.76 ± 8.48	22.64 ± 8.59	2.82 ± 1.25	2.43 ± 1.39	E	G	3	①②③
Liang D. 2016^[47]^	40	40	22/18	21/19	34.32 ± 2.43	32.65 ± 2.35	-		D	G	1	①②③
Ren Y.H. 2016^[48]^	40	40	-						C	G	3	①②③
Guo Z.H. 2016^[49]^	40	40	22/18	24/16	26.30 ± 4.90	27.10 ± 4.60	4.30 ± 1.90	4.10 ± 1.70	A	G	1	①②⑤
Yu J. B. 2015^[50]^	67	67	34/33	35/32	46.80 ± 4.50	47.70 ± 4.10	7.70 ± 3.76	7.90 ± 3.93	A	G	3	①②③
Wei Y.D. 2015^[51]^	70	70	31/39	33/37	36.51 ± 7.68	35.72 ± 6.91	21.16 ± 17.21	19.67 ± 15.72	A	G	4	①②⑤
Li X.M. 2015^[52]^	70	65	102/98		32.40 ± 17.40		4.70 ± 5.60		D	G	3	①②⑤
Du G.M. 2015^[53]^	32	31	15/17	16/15	25.22 ± 6.57	25.32 ± 7.82	20.16 ± 11.80	19.00 ± 14.84	A	G	3	①②③
	34		17/17		27.82 ± 8.43		24.11 ± 11.29		B			
Shan W.H. 2015^[54]^	63	63	34/29	32/31	26.90 ± 1.40	26.50 ± 1.80	6.40 ± 1.10	7.20 ± 1.50	C	D	6	①②
Wang Y.J. 2013^[55]^	53	52	70/87		-		-		C	A	3	①②③
Liu W.C. 2013^[56]^	37	36	18/19	20/16	27.60 ± 5.83	29.00 ± 6.83	7.20 ± 3.15	8.10 ± 3.47	C	G	3	①②③
Jiang P. 2013^[57]^	46	46	22/24	20/26	19.50 ± 12.80	19.70 ± 12.60	8.30 ± 5.10	8.50 ± 4.90	A	G	3	①②⑥
Hao Z.F. 2013^[58]^	80	80	126/114		31.75 ± 11.10		6.78 ± 4.84		C	G	6	①②③
Su Z.X. 2012^[59]^	60	40	28/32	21/19	-		-		C	G	4	①②③
Lin C.S. 2012^[60]^	50	50	-		-		-		E	G	3	①②③
Li D.S. 2012^[61]^	54	54	71/37		28.90 ± 4.60		1.10 ± 0.60		C	D	2.5	①②
Zhao L.L. 2011^[62]^	60	65	24/36	29/36	21.30 ± 6.83	23.70 ± 8.33	2.50 ± 1.42	2.20 ± 1.82	A	G	3	①②③
Zhang H.B. 2011^[63]^	40	40	51/69		28.60 ± 8.00		2.70 ± 2.61		B	G	3	①②
Wang J.M. 2010^[64]^	76	72	41/35	40/32	30.00 ± 6.17		1.50 ± 0.66		C	D	6	①②
Qin Y.Q. 2010^[65]^	33	30	18/15	19/11	29.31 ± 2.34	14.20 ± 2.37	28.30 ± 4.05	13.32 ± 3.67	C	G	2.5	①②
Chen F. 2010^[66]^	45	40	76/52		32.50 ± 8.83		6.50 ± 3.29		B	G	4	①②③
Zhang X.P. 2009^[67]^	62	31	33/29	17/14	27.50 ± 7.67	27.60 ± 8.00	3.60 ± 1.79	3.40 ± 1.47	A	G	6	①②③
Liu C.J. 2009^[68]^	18	14	21/11		38.00 ± 9.67		-		A	G	3	①②③
Yang M.H. 2005^[69]^	34	22	26/50		35.50 ± 4.67		-		A	G	2	①②③
Wen D.H. 2005^[70]^	55	43	24/31	18/25	20.60 ± 4.33	23.00 ± 4.33	1.60 ± 1.29	1.50 ± 1.11	B	G	6	①②③
Shen Y. 2004^[71]^	42	32	31/43		28.00 ± 7.83		-		A	G	4	①②

Abbreviations of this table are shown as follow:

Groups: e.g. = experimental group, CG = control group.

Interventions: A = VCP combined with RT, B = BTC combined with RT, C = QT combined with RT, D = BP combined with RT, E = TP combined with RT, F = QP combined with RT, G = RT.

Outcomes: ① total effective rate; ② good improvement rate; ③ adverse reactions; ④ skin lesion pigment score; ⑤ area of lesion; ⑥ life quality score according to Dermatosis Life Quality Index Scale.

### 3.2. Quality assessment

**Figure 2.**
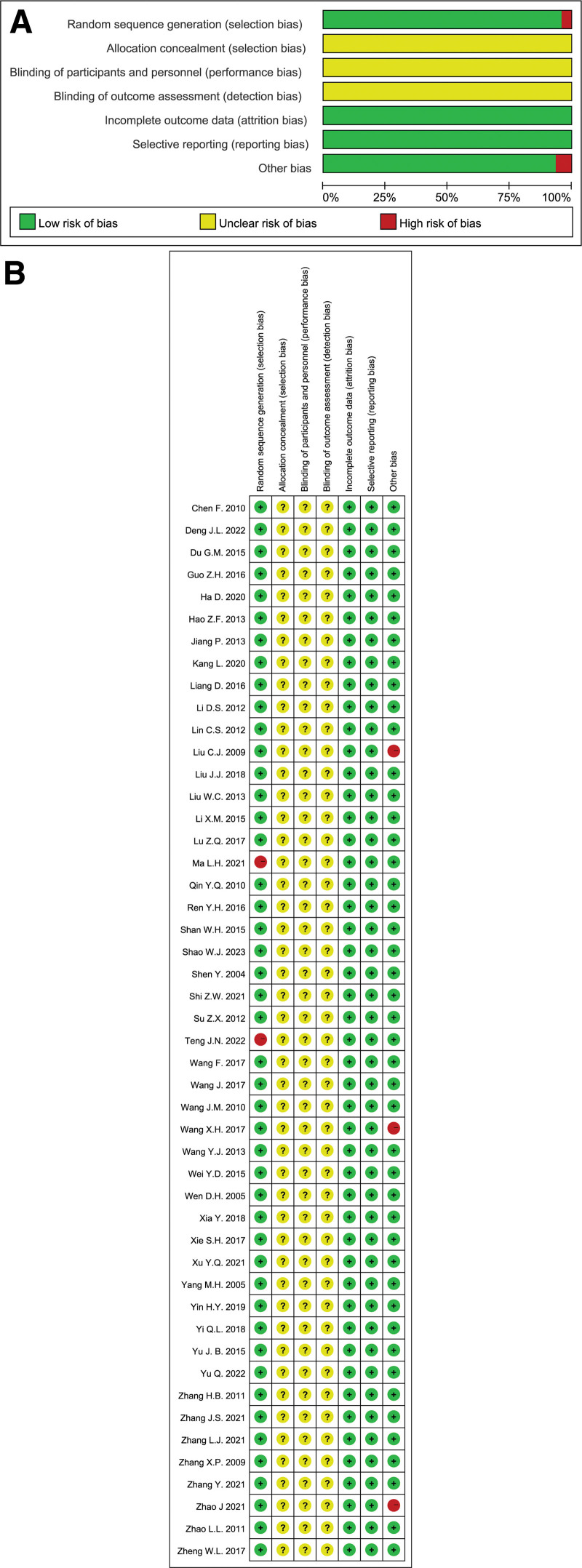
Quality assessment of the included literature. The levels of risk were represented by the color of the bars and circles. Red was regarded as high-risk; yellow was regarded as unclear; and green was regarded as low-risk. We used the author name and publication year to represent the relevant literature.

A quality assessment of the included articles is shown in Figure 2. The results indicated that most of the studies were classified into unclear and low-risk groups. Therefore, the general results of all studies were acceptable.

### 3.3. Transitivity assessment

In order to verify the transitivity of all the included studies, we choose to evaluate the similarity of each study from the perspectives of sex, age, disease course and the duration of each intervention treatment. Firstly, the proportion of male patients was used to evaluate the sex differences between the groups directly compared. The results set out in Supplemental Figure 1 (a), http://links.lww.com/MD/K65 showed that most of the point estimated values of the average total evaluation of each group directly compared varied from 40% to 60%, and the point estimated values of each group were comparable (50%). Secondly, we also investigated the age deviation of participants in each study. The results set out in Supplemental Figure 1 (b), http://links.lww.com/MD/K65 showed that the point estimate of the total average age of each group was between 20 and 40 years old, and the point estimated values of each group were comparable (30 years old). Thirdly, we also analyzed the disease course of the participants and the duration of treatment. Although the disease course and the duration of treatment in each group were slightly different, the point estimate of the total average on the disease course and duration of treatment of each group was within 5 to 10 years and 2.5 to 5 months, respectively, as shown in Supplemental Figure 1 (c), http://links.lww.com/MD/K65 (disease course) and Supplemental Figure 1 (d), http://links.lww.com/MD/K65 (duration of treatment). In conclusion, all the included studies have good transitivity, which can be analyzed by network meta-analysis to compare the efficacy and safety of various Chinese patent medicines in the treatment of vitiligo.

### 3.4. Network diagram

**Figure 3.**
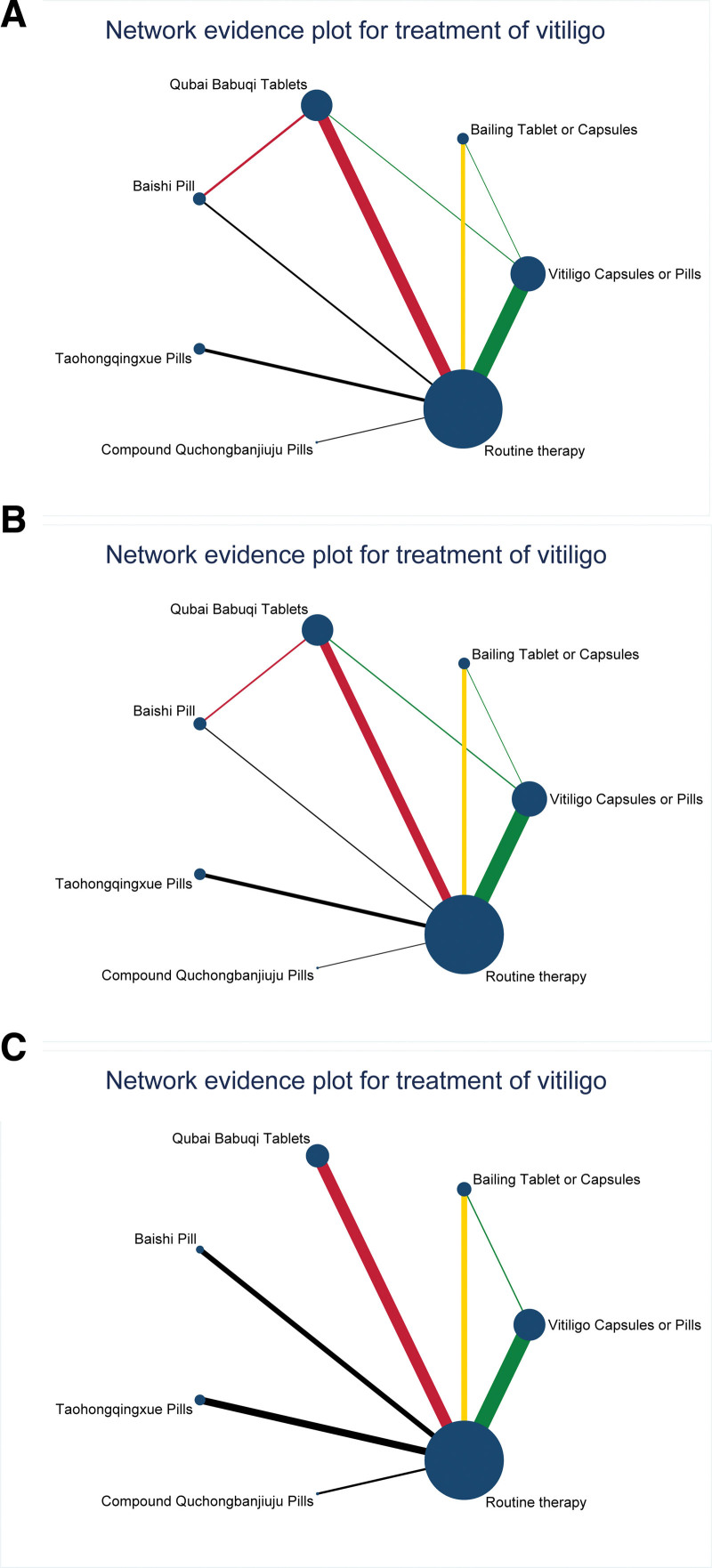
Network evidence plot of primary indicators. Primary indicators were total effective rate (A), good improvement rate (B), and adverse reactions (C). The line between 2 dots directly compares the 2 interventions, the thickness of which indicates the number of randomized controlled trials involved. The size of the dots indicates the sample size of the intervention, and no line between the 2 dots indicates that there was no direct comparison between the 2 measures and that the efficacy of the 2 measures had to be compared indirectly.

**Figure 4.**
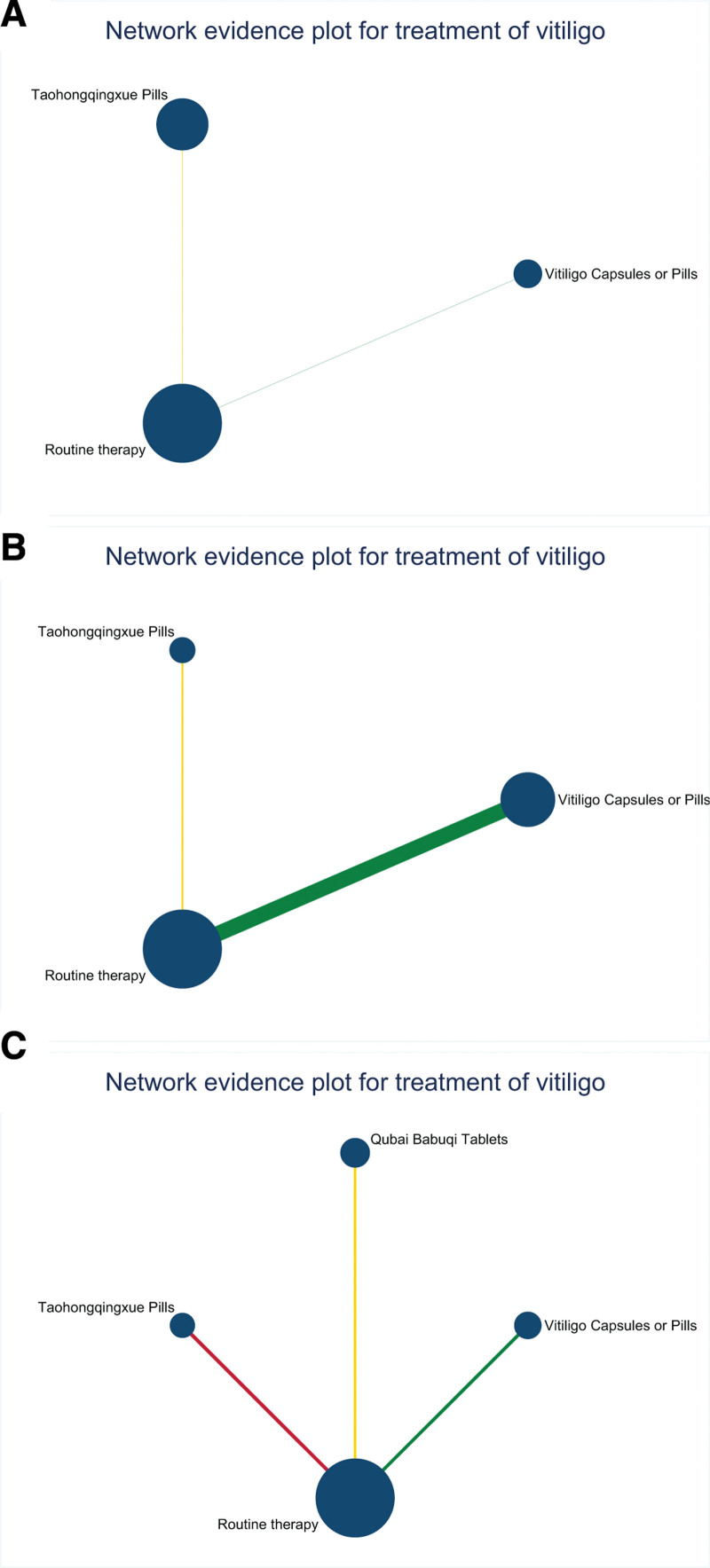
Network evidence plot of secondary indicators. Secondary indicators were skin pigment score (A), lesion area (B), and quality-of-life score (C). The line between the 2 dots directly compares the 2 interventions, the thickness of which indicates the number of randomized controlled trials involved. The size of the dots indicates the sample size of the intervention, and no line between the 2 dots indicates that there was no direct comparison between the 2 measures and that the efficacy of the 2 measures had to be compared indirectly.

Forty-eight RCTs for 6 CPMs and routine therapy reported total efficiency and good improvement rates. Thirty RCTs of 6 target drugs and routine therapy reported adverse reactions. Three RCTs for 2 target drugs and routine therapy reported a skin lesion pigment score. Five RCTs for 2 target drugs and routine therapy reported a lesion area. Lastly, 3 RCTs for 3 target drugs and routine therapy reported a quality-of-life score (Figs. 3 and 4). The sample size of QT and VCP is relatively large, while the sample size of other CPMs is relatively small. The included studies included direct comparisons of the efficacy and safety of all 6 CPMs and conventional measures, as well as the efficacy of VCP, BCT, and QT and the safety of VCP and BCT.

### 3.5. Inconsistency test for closed loops

**Figure 5.**
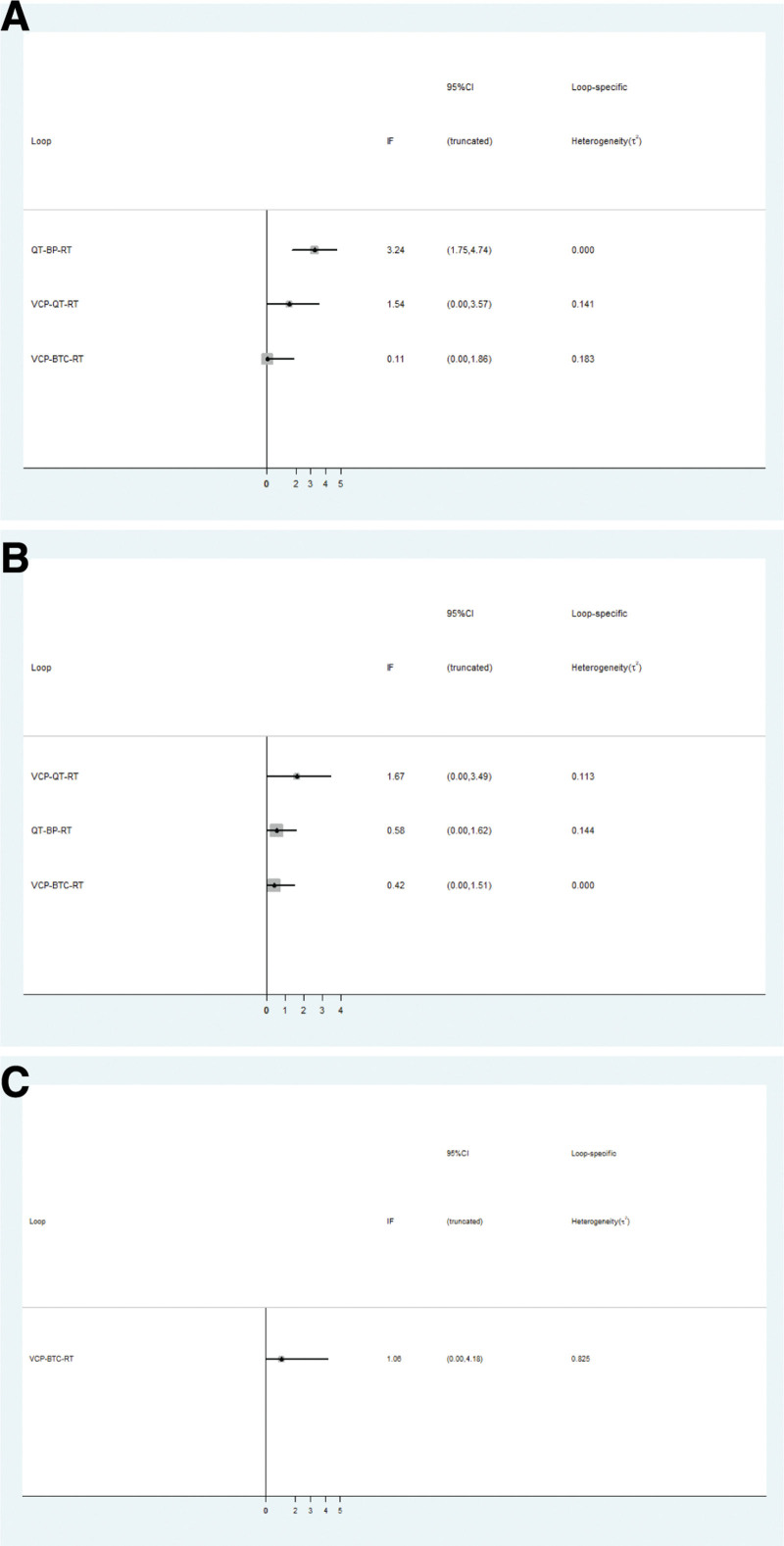
Inconsistency test of the primary indicators. The primary indicators included total effective rate (A), good improvement rate (B), and adverse reactions (C). In this figure, we estimated the inconsistency of the closed loops according to the results. If the IF values were in close proximity to zero or its 95% CI included zero, and t^2^ values were >0.05, the consistency of the direct comparison was good. Otherwise, the consistency was poor.

The major outcomes of the total effective and good improvement rates for the 6 CPMs constituted 3 quadrilateral closed loops. Inconsistency and heterogeneity tests were performed using IFs. The results of the IFs, 95% CIs, and t^2^ statistics (loop-specific heterogeneity) are presented in Figure 5. The consistency of 1 loop (QT-BP-RT) was not satisfactory [IF: 3.24; 95% CI: (1.75, 4.74); t^2^: 0.000], but the remaining results were satisfactory [IF: 0.11–1.67; 95% CI: (0.00, 3.49); t^2^: 0.000–0.144], which indicated that direct and indirect comparisons had little influence on the results of the analysis, and that there may be a slight deviation in the results of the NMA. The adverse effects of 6 CPMs constituted 1 quadrilateral closed loop. However, its consistency was satisfactory [IF: 1.06; 95% CI: (0.00, 4.18); t^2^: 0.825]. As the remaining outcomes did not form a loop, there was no need to conduct an inconsistency test.

### 3.6. Network meta-analysis

Before conducting the network meta-analysis, we used the Cochrane Q test to evaluate heterogeneity among the included studies. The results showed that the following results for the *P* value and I^2^ of the test of each outcome index: total effective rate (*P* < .00001, I^2^ = 63%), good improvement rate (*P* < .0001, I^2^ = 51%), adverse reactions (*P* = .010, I^2^ = 26%), skin lesion pigment score (*P* = .010, I^2^ = 77%), lesion area (*P* = .07, I^2^ = 57%) and quality-of-life score (*P* = .84, I^2^ = 0%). Although the I^2^ values for most of the studies was more than 50, and the test *P* values for some studies were <.01, the I^2^ value of each outcome index was approximately between 50% and 75%, which shows that although there is heterogeneity among the studies, it is within an acceptable range, and the random effects model can be used for network meta-analysis.

In our study, the main efficacy indicators were the total effective and good improvement rates, and the incidence of adverse reactions was the main safety index. In addition, the skin lesion pigment scores, lesion areas, and quality of life scores were selected as secondary outcome indicators to evaluate the efficacy of CPM.

#### 3.6.1. Total effective rate and good improvement rate.

**Figure 6.**
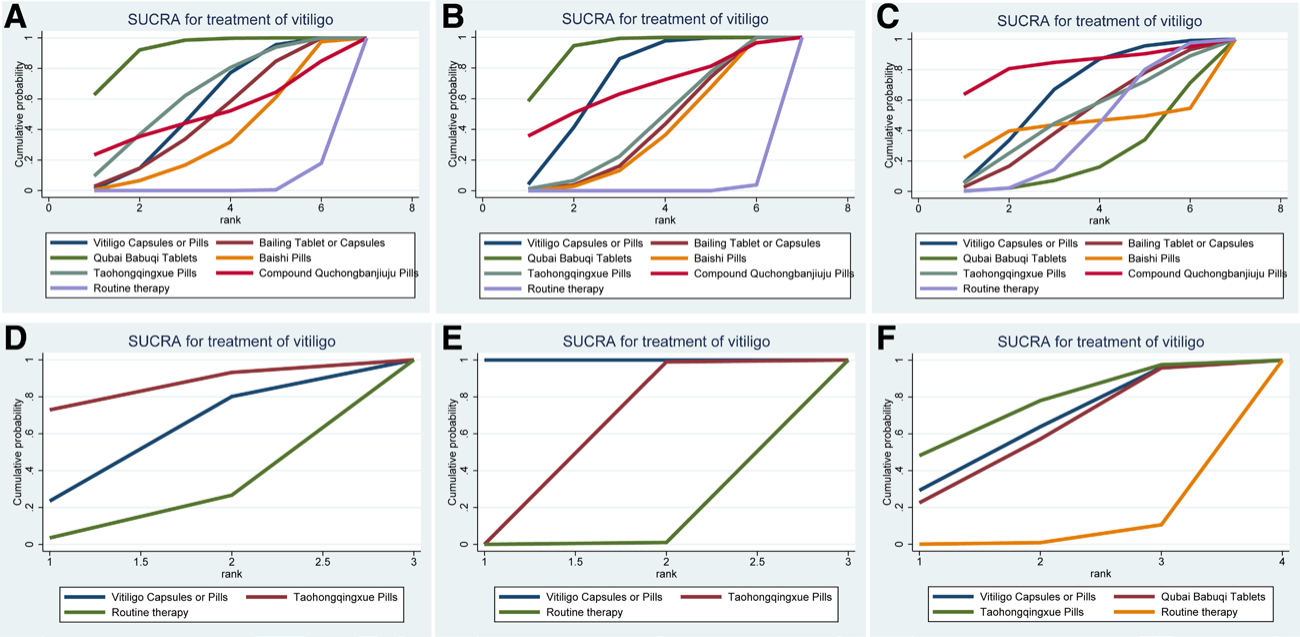
Cumulative probability of all outcome indicators. In Figure 6A, 6B, and 6C, the abscissa represented the ranking order from first to seventh, and the ordinate represented the cumulative probability of the 7 interventions in each ranking. In Figure 6D and 6E, the abscissa represented the ranking order from first to third, and the ordinate represented the cumulative probability of the 3 interventions in each ranking. In Figure 6F, the abscissa represented the ranking order from first to fourth, and the ordinate represented the cumulative probability of the 4 interventions in each ranking.

**Figure 7.**
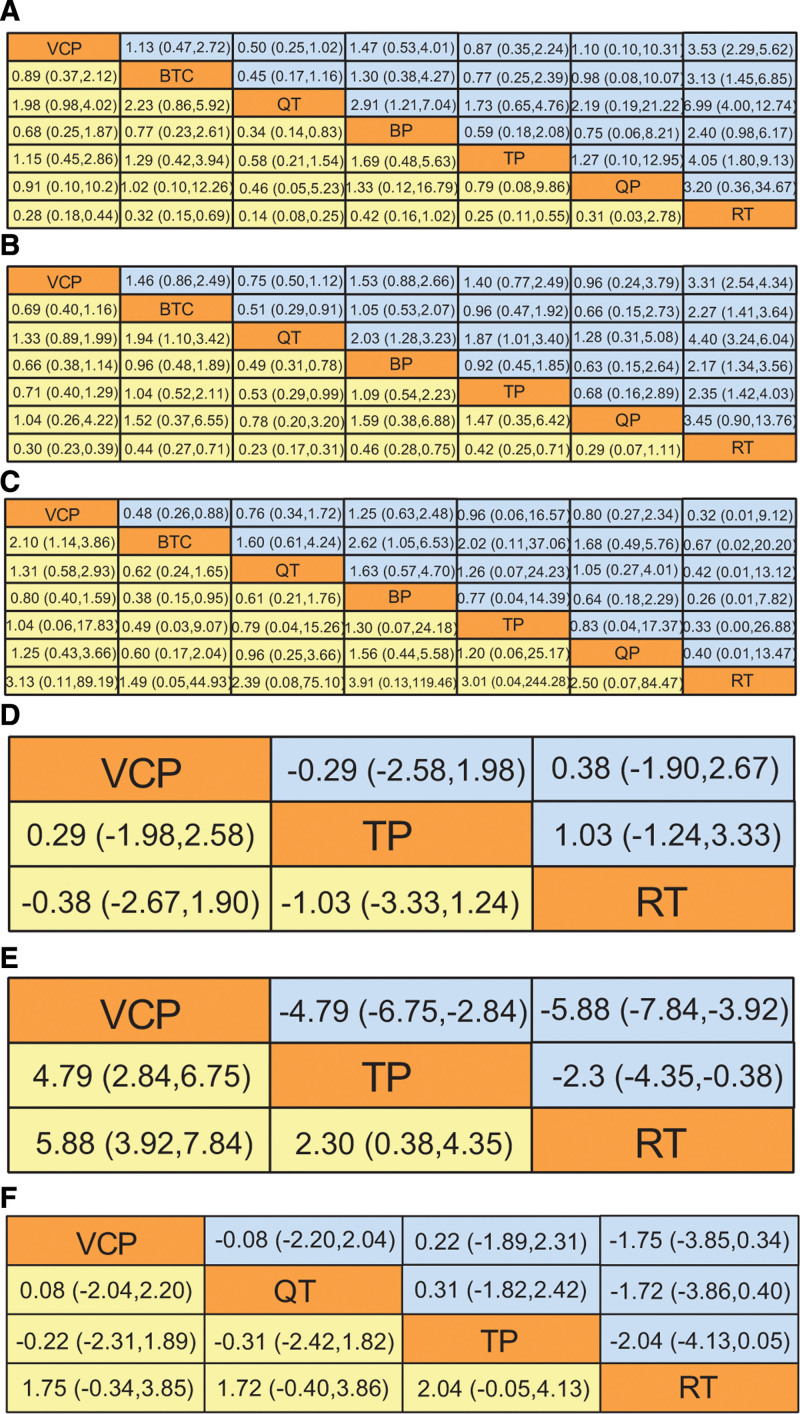
League table of results of the network meta-analysis for all outcome indicators. In Figure 7A, 7B, and 7C, the 7 columns showed the OR values and 95% CI for each of the 7 interventions. Here, the OR value in the yellow box was reciprocal to the OR value in the corresponding blue box. In Figure 7D and 7E, the 3 columns showed the SMD values and 95% CI for each of the 3 interventions. Here, the SMD value in the yellow box was opposite to that in the corresponding blue box. In Figure 7F, the 4 columns showed the SMD values and 95% CI for each of the 4 interventions. Here, the SMD value in the yellow box was opposite to that in the corresponding blue box.

In terms of the total effective rate, compared with traditional Western medicine, QT had the best efficacy (OR = 6.99; 95% CI (4.00, 12.74); *P* < .05]. The second most effective drug was TP (OR = 4.05; 95% CI (1.80, 9.13); *P* < .05] and the third was VCP (OR = 3.53; 95% CI (2.29, 5.62); *P* < .05]. The cumulative probability plot and league table of the efficacies of the 6 CPMs are shown in Figures 6A and 7B. According to the SUCRA values, the efficacy of the 6 CPMs was in the order of QT (92.18%) > TP (63.81%) > VCP (55.53%) > QP (50.72%) > BTC (49.01%) > BP (35.69%) (Supplemental Table 1, http://links.lww.com/MD/K66). In terms of the improvement rate, compared with traditional Western medicine, QT had the best efficacy (OR = 4.40; 95% CI (3.24, 6.04); *P* < .05]. QP was second (OR = 3.45; 95% CI (0.90, 13.76); *P* < .05], and VCP was third (OR = 3.31; 95% CI (2.54, 4.34); *P* < .05]. The cumulative probability plots and league tables of the efficacies of the 6 CPMs are shown in Figures 6B and 7B. According to the SUCRA values, the efficacy of the 6 CPMs followed the order: QT (92.05%) > VCP (71.50%) > QP (66.60%) > TP (42.95%) > BTC (39.66%) > BP (36.60%) (Supplemental Table 1, http://links.lww.com/MD/K66). In the analysis of total effective rate, the sum of deviation parameters was 94.33, which approximated the total number of research arms amounting to 97. In the analysis of good improvement rate, the sum of deviation parameters was 99.16, which approximated the total number of research arms amounting to 97. It showed that the model used was a good fit and the results are reasonable.

#### 3.6.2. Adverse reactions.

The adverse reactions reported in the 48 target studies included skin lesions (erythema, papules, and blisters) and abnormal pain sensations (itching and dryness). In addition, gastrointestinal reactions and abnormalities in the liver and kidney functions were also observed. According to the results of NMA, in comparison with routine therapy, the safety of QP [OR = 4.40; 95% CI (0.01, 13.47)], VCP [OR = 0.32; 95% CI (0.01, 9.12)], TP [OR = 0.33; 95% CI (0.00, 26.88)], BTC [OR = 0.67; 95% CI (0.02, 20.20)], BP [OR = 0.26; 95% CI (0.01, 7.82)], and QT [OR = 0.42; 95% CI (0.01, 13.12)] was not significantly different (*P* > .05). The cumulative probability plot and league table of the 7 interventions according to the incidence of adverse reactions are shown in Figures 6C and 7C. In terms of the SUCRA values, the order of the CPMs was as follows: QP (83.72%) > VCP (64.66%) > TP (49.16%) > BTC (48%) > BP (42.74%) > routine therapy (39.89%) > QT (21.83%) (Supplemental Table 1, http://links.lww.com/MD/K66). These results indicate that QP performed the best while QT performed the worst. In this analysis, the sum of deviation parameters was 57.37, which was approximate to the total number of research arms amounting to 60. It showed that the model used was a good fit and the results are reasonable.

#### 3.6.3. Skin lesion pigment score, lesion area, and quality-of-life score.

In our study, 3 articles were included that measured lesion pigment score and quality-of-life score, and 4 were included that measured the lesion area. The cumulative probability plot and league table of changes in the lesion pigmentation score, lesion area, and quality-of-life score are presented in Figures 6D and 7D. In comparison with routine therapy, VCP [SMD = 0.38; 95% CI (−1.9, 2.67)] and TP [SMD = 1.03; 95% CI (−1.24, 3.33)] showed no significant difference (*P* > .05). According to the SUCRA values, the results showed that TP (83.06%) was better than VCP (51.81%) (Supplemental Table 1, http://links.lww.com/MD/K66). The cumulative probability plot and league table of changes in the lesion area are shown in Figures 6E and 7E. In comparison with routine therapy, VCP [SMD = −5.88; 95% CI (−7.84, −3.92); *P* < .05] was slightly better than TP [SMD = −2.30; 95% CI (−4.35, −0.38); *P* < .05]. According to the SUCRA values, the efficacy of VCP (100%) was higher than that of TP (49.49%) (Supplemental Table 1, http://links.lww.com/MD/K66). The cumulative probability plot and league table of changes in the quality-of-life scores are presented in Figures 6F and 7F. In comparison with routine therapy, VCP [SMD = −1.75; 95% CI (−3.85, 0.34)], QT [SMD = −1.72; 95% CI (−3.86, 0.40)], and TP [SMD = −2.04; 95% CI (−4.13, 0.05)] had no significant differences (*P* > .05). According to the SUCRA values, the results showed that TP (74.59%) > QT (63.08%) > VCP (58.50%) (Supplemental Table 1, http://links.lww.com/MD/K66).

### 3.7. Cluster analysis of efficacy and security

**Figure 8.**
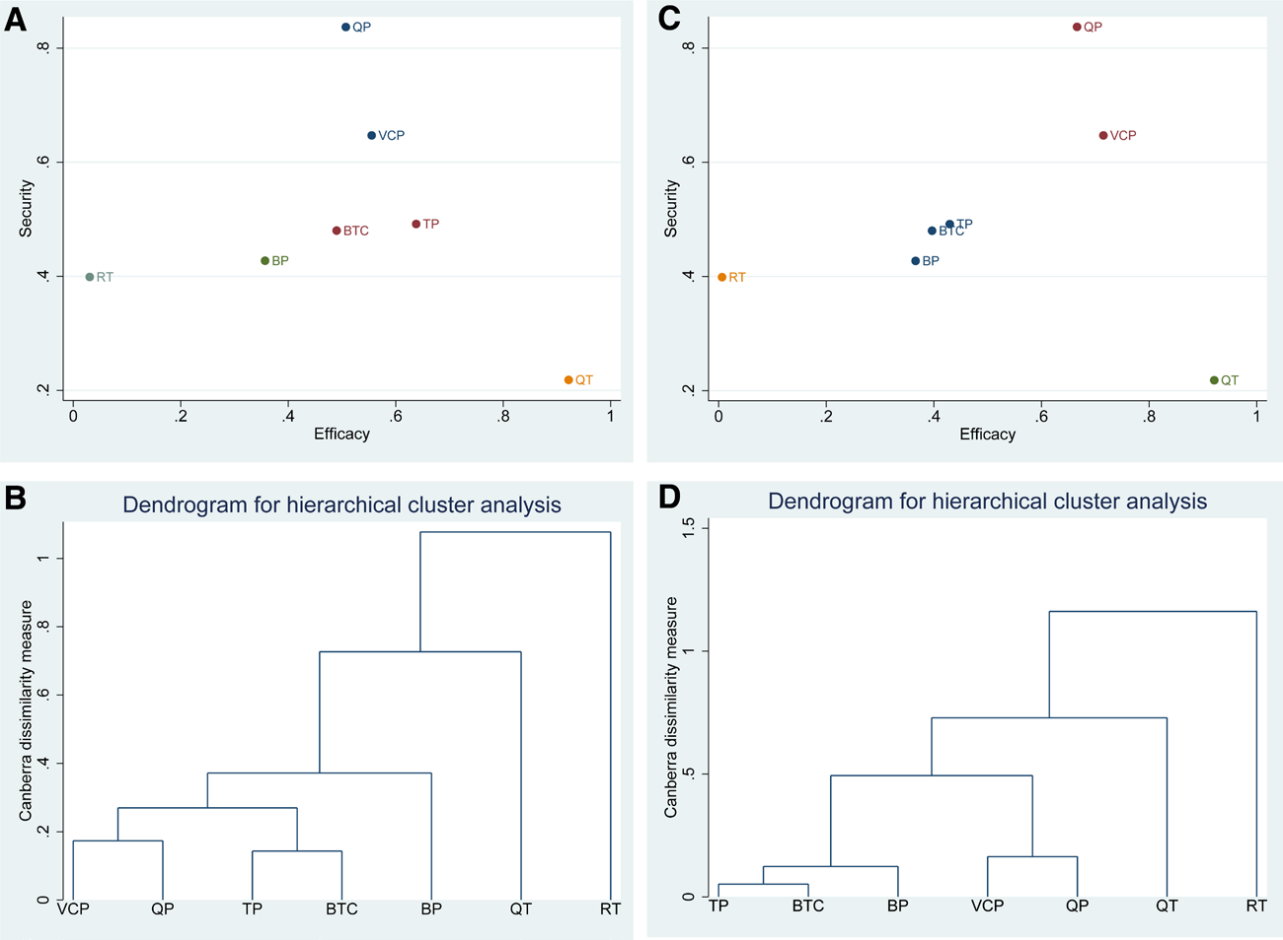
Scattergram and dendrogram for cluster analysis. We adopted the average linkage as the best linkage method and the Canberra distance as the best distance metric. In (A), the abscissa labeled efficacy represents the total effective rate ranging from 0 to 1, and the ordinate named security represents the adverse effect ranging from 0 to 1. The color of the points indicates the classification. In (B), the abscissa indicates the 7 interventions, and the ordinate indicates their distance dissimilarity. In (C), the abscissa labeled efficacy represents good improvement rate ranging from 0 to 1, and the ordinate labeled security represents the adverse effect ranging from 0 to 1. The color of the points indicates the classification. In (D), the abscissa indicates the 7 interventions, and the ordinate indicates their distance dissimilarity.

Based on the results of the NMA, the total effective and good improvement rates were analyzed using cluster analysis with the incidence of adverse reactions. The results are shown in the scattergram and dendrogram in Figure 8, respectively. The results of the total effective rate and incidence of adverse reactions were grouped into 5 groups after cluster analysis as follows: routine therapy with lower efficacy and safety; BP and BCT with lower efficacy and medium safety; TP with medium efficacy and safety; VCP and QP with medium efficacy and higher safety; and QT with higher efficacy and lower safety. The results of the good improvement rate and incidence of adverse reactions were grouped into 4 groups after cluster analysis as follows: routine therapy as a comparison with low efficacy and safety; BP, BTC, and TP with medium efficacy and safety; VCP and QP with medium efficacy and high safety; and QT with high efficacy and low safety.

### 3.8. Publication bias analysis

**Figure 9.**
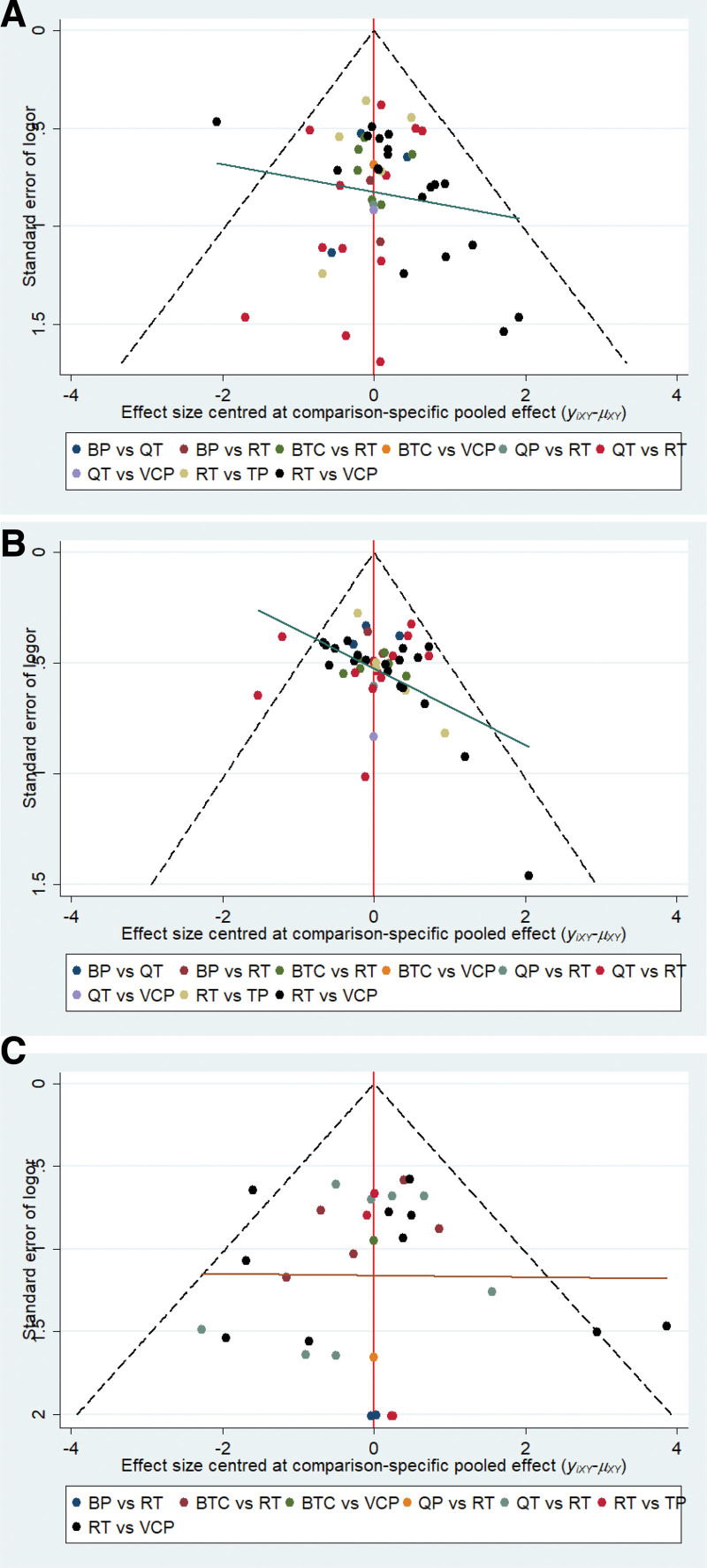
Comparison corrected funnel plot of primary indicators. The primary indicators were total effective rate (A), good improvement rate (B), and adverse reactions (C). The dots in this figure represent all the direct comparisons. If publication bias was low, most dots would be located inside the funnel and approximately symmetrically distributed across 2 sides of the midline. In addition, the angle of the midline and the correction auxiliary line would be small.

A comparison-corrected funnel plot of the major outcomes is shown in Figure 9. Figure 9A and B have good symmetry. Most studies were roughly symmetrically distributed to 2 sides of the midline.^[72]^ Additionally, the angle between the correction auxiliary line and the midline is small, indicating less possibility of publication bias. Figure 9C has general symmetry, and most studies were roughly symmetrically distributed to the left of the midline, indicating a small sample effect. There is a certain angle between the correction auxiliary line and the midline, which may result from the low accuracy of the research.^[73]^

### 3.9. Evidence grade for NMA

A grading tool was adopted to evaluate the evidence level of comparisons and the rank of treatments, which was carried out in 5 aspects: study limitation, indirectness, heterogeneity, inconsistency, imprecision, and publication bias. The results are presented in the grade table, and the reasons for downgrading are listed in Table 3. These findings indicate that the indirect comparison between VCP and TP was high regarding the total effective rate. The rank of the treatments was moderate because there was no significant difference in the inconsistency model testing (*P* < .05). Concerning the improvement rate, the comparison between VCP and QT, BP, and TP was high. The ranking of the treatments was also high. There was no strong evidence regarding adverse effects, and the rank of treatments was low because of heterogeneity and publication bias.

**Table 3:** Grade table for NMA evidence.

	Total effective rate			Good improvement rate			Adverse reactions		
	Nature of the evidence	Nature of the evidence	Nature of the evidence	Nature of the evidence	Nature of the evidence	Nature of the evidence	Nature of the evidence	Nature of the evidence	Nature of the evidence
Comparison									
AB	Mixed	Moderate	③	Mixed	Moderate	④	Mixed	Moderate	③
AC	Mixed	Moderate	③	Mixed	High		Indirect	Moderate	⑤
AD	Indirect	Moderate	⑤	Indirect	High		Indirect	Moderate	⑤
AE	Indirect	High		Indirect	High		Indirect	Moderate	⑤
AF	Indirect	Moderate	①	Indirect	Moderate	①	Indirect	Low	①⑤
AG	Mixed	Low	⑤⑥	Mixed	Low	⑤⑥	Mixed	Low	⑤⑥
BC	Indirect	Moderate	⑤	Indirect	Moderate	⑤	Indirect	Moderate	⑤
BD	Indirect	Moderate	⑤	Indirect	Moderate	⑤	Indirect	Moderate	③
BE	Indirect	Moderate	⑤	Indirect	Moderate	⑤	Indirect	Moderate	⑤
BF	Indirect	Low	①⑤	Indirect	Low	①⑤	Indirect	Low	①⑤
BG	Mixed	Moderate	⑤	Mixed	Moderate	⑤	Mixed	Moderate	⑤
CD	Mixed	Low	④⑤	Mixed	Moderate	③	Indirect	Moderate	⑤
CE	Indirect	Moderate	⑤	Indirect	Moderate	⑤	Indirect	Moderate	⑤
CF	Indirect	Low	①⑤	Indirect	Low	①⑤	Indirect	Low	①⑤
CG	Mixed	Low	⑤⑥	Mixed	Low	⑤⑥	Mixed	Low	⑤⑥
DE	Indirect	Moderate	③	Indirect	Moderate	③	Indirect	Moderate	⑤
DF	Indirect	Low	①⑤	Indirect	Low	①③	Indirect	Low	①⑤
DG	Mixed	Moderate	⑤	Mixed	Moderate	⑤	Mixed	Moderate	⑤
EF	Indirect	Low	①⑤	Indirect	Low	①⑤	Indirect	Low	①⑤
e.g.	Mixed	Moderate	⑤	Mixed	Moderate	⑤	Mixed	Moderate	⑤
FG	Mixed	Low	①⑤	Mixed	Low	①⑤	Mixed	Low	①⑤
Rank of treatments		Moderate	④		High			Low	③⑥

The grade of evidence was estimated according to the following 5 aspects: study limitation, indirectness, heterogeneity, inconsistency, imprecision and publication bias, which are respectively numbered from 1 to 5 as the reasons for degradation in this table.

A = VCP combined with RT, B = BTC combined with RT, C = QT combined with RT, D = BP combined with RT, E = TP combined with RT, F = QP combined with RT, G = RT.

## 4. Discussion

### 4.1. Advantages of CPMs in treating vitiligo

Vitiligo is a dermal depigmentation disease that is difficult to treat. It has a considerable negative impact on patients physically, mentally, and socially, seriously affecting quality of life.^[74]^ Recent studies have shown that some natural drugs, such as Ecliptae Herba, Polygoni Multiflori Radix Praeparata, and Rehmanniae Radix Praeparata, can be used to treat depigmentation dermal diseases. Their extracts can significantly improve tyrosinase activity in the skin, but the specific compositions and mechanisms of action remain unclear.^[75]^ Treatment of vitiligo is typically long, often requiring 3 to 6 months or more of treatment. Traditional oral medicines and medicines administered by injection cannot be used long-term because of their adverse effects.^[76]^ Phototherapy may cause serious damage to the localized skin at high doses, and erythema and blisters often occur.^[77]^ In contrast, traditional Chinese medicine has considerable advantages in the treatment of vitiligo. However, the preparation and administration of traditional herbal preparations are complicated, and patient compliance is poor. Moreover, despite their suitability for individualized diagnosis and treatment, it is difficult to accurately estimate their efficacy due to a lack of unified standards.^[78]^ Preparation of CPMs is standardized, administration is straightforward, and instructions are easy for patients to follow, and CPMs have few side effects; therefore, patient compliance with CPMs is good. For these reasons, CPMs are suitable as an oral preparation for the long-term treatment of vitiligo.

### 4.2. Design features of our research

Currently, most studies on the treatment of vitiligo with CPMs involve small samples, are unicentric, and are based on low report and evaluation quality. To date, there is a lack of robust evidence on the efficacy and safety of various common CPMs for treating vitiligo. This study aimed to compare the efficacy and safety of 6 common CPMs used for the treatment of vitiligo using a Bayesian network meta-analysis to provide robust evidence supporting their long-term use as oral treatment for vitiligo. A total of 48 RCTs were included, with a total sample size of 4446 cases. Similar to other RCTs, the following measures were used as the final outcomes: estimated ratio of skin lesion recovery as the standard to assess curative effect; the total effective and good improvement rates were used as the final indicators to compare the curative effects; the total effective rate is the sum of the cured rate, marked effective rate, and effective rate, whereas the good improvement rate is the sum of the cured rate and marked effective rate.^[79]^ The degree of depigmentation and the area of skin lesions in vitiligo are also important indicators of the severity of vitiligo and were used as secondary indicators of efficacy in our study. Patients with vitiligo suffer considerably in their daily and social lives, reducing their quality of life. Therefore, quality of life scores in patients with vitiligo were also used as a secondary efficacy index. For the safety evaluation, the incidence of adverse reactions was selected as the main indicator.^[80]^

### 4.3. Findings and explanations

Our evaluation showed that QT had the highest efficacy based on the total effective rate and cure rate; however, it had a weak safety profile, potentially because of the adverse effects caused by its component plumbagin. Although the efficacy of QP, which has a similar composition, is slightly inferior to that of QT, QP is considerably safer. The pathogenesis of vitiligo is closely related to the apoptosis of melanocytes, induced by various signaling pathways.^[81–83]^ Previous studies have shown that the PI3K/Akt/mTOR signaling pathway plays an important role in regulating the functions of many cell types, including melanocytes, consisting of proliferation, survival, and migration.^[84,85]^ Basic fibroblast growth factors (BFGFs) have been shown to promote melanocyte migration by regulating PI3K/Akt, Rac1, FAK, JNK, and ERK activities.^[86]^ Modern pharmacological studies have indicated that plumbagin, the main active component of Plumbaginaceae, has noticeable pharmacological effects, such as anti-tumor,^[86]^ antibacterial,^[87]^ anti-inflammatory,^[88]^ neuroprotective,^[89]^ and myocardial cell protection.^[90]^ Its underlying molecular mechanism is related to the regulation of PI3K/Akt/mTOR, PI3K/Akt/GLUT, and other signaling pathways.^[91]^ Therefore, the active components of plumbagin may promote the proliferation, survival, and migration of melanocytes to achieve the desired repigmentation effect. However, plumbagin exhibits considerable cytotoxicity,^[92]^ and the incidence of adverse reactions to QT is high. Therefore, QT may be appropriate for controlling the progression of vitiligo in the short-term. After disease remission, the compound QP should be recommended as a long-term medication to achieve repigmentation. The total effective rate of VCP was better than that of TP, BTC, and BP. The incidence of adverse reactions was also lower, indicating its suitability for the long-term treatment of patients with vitiligo. In terms of efficacy, the greatest efficacy was found for TP followed by BTC and then BP, and adverse reaction rates for these interventions were lower than those of conventional Western medicine.

In our study, cluster analysis was conducted according to the analysis results of the main outcome indicators, and the efficacy and safety of 6 types of CPMs and traditional western medicines were classified. According to the results, VCP and QP may have relatively good efficacy and very high safety. QP, like QT, contains a common effective pharmaceutical ingredient called Vernonia anthelmintica, which has a beneficial therapeutic effect on vitiligo. Previous studies have shown that flavonoids such as isorhamnetin, isocarthamidin, kaempferide, fisetin, liquiritigenin, and chalcone derivatives such as butein, isoliquiritigenin obtained from the ethanol extract of Vernonia anthelmintica can promote melanin synthesis of human primary melanocytes and B16F10 melanoma cells by activating p38 mitogen-activated protein kinase.^[93,94]^ In addition, recent research shows that the isolated cynarine can also promote the phosphorylation of p38 mitogen-activated protein kinase to increase the intracellular melanin level and tyrosinase activity without cytotoxicity.^[95]^ Vernonia anthelmintica has been shown to have broad prospects for application in the treatment of vitiligo. Compared with BTC, BP, TP and TP, VCP has a special medicinal component called Zaocys dhumnades. Although similar drugs such as Zaocys dhumnades and snake slough are often used in the treatment of vitiligo in traditional Chinese medicine, the specific mechanisms of drug action remain unclear. This study speculated that Zaocys dhumnades is the key drug component with remarkable curative effect on VCP. Previous studies on the metabonomics characteristics of amino acids in the plasma of patients with vitiligo have shown that the reduction of arginine, glycine, lysine, histidine and ornithine can damage melanocytes and lead to vitiligo lesions on the skin surface of patients.^[96]^ However, the modern known pharmacological research of Zaocys dhumnades shows that its snake muscle is rich in 17 kinds of amino acids, including glycine, lysine, arginine, and histidine.^[97]^ Therefore, it can be inferred that the treatment of vitiligo by Zaocys dhumnades may be related to improving the amino acid metabolism in patients, thus restoring the function of melanocytes. Further studies are required to investigate the specific mechanism involved. Therefore, compared with BTC, BP and TP, VCP has obvious advantages in the treatment of vitiligo.

### 4.4. Advantages and limitations

In the previous search, there was no network meta-analysis study on the efficacy and safety evaluation of CPMs in the treatment of vitiligo, so this may be the first research in the field. Based on the hypothesis of homogeneity, similarity, and consistency in methodology, Bayesian statistical methods were used for analysis, which ensured the reliability of the analysis results, thus providing more robust evidence for the long-term use of Chinese patent medicine in vitiligo treatment. Currently, traditional Chinese medicine treatments for vitiligo are diverse. Our study only selected common Chinese patent drugs for comparison to provide evidence for the selection of long-term oral drugs in clinical practice; therefore, it failed to fully reflect the diversity of traditional Chinese medicine vitiligo treatments, and the practicability of the network meta-analysis results may be biased. Considering that researchers selectively reported positive results, the quality of evidence of the included studies was low, and there was a risk of exaggerated results. Moreover, the heterogeneity of some studies was high. The original studies were mostly in Chinese due to the use of CPM as the object of comparison in our study. Higher-quality clinical studies worldwide are needed to supplement these findings to better guide the clinical use of Chinese patent medicine in this field. Other factors, such as the course of treatment, impacted the conclusions. Therefore, clinical decision-makers should consider various factors when applying these conclusions to improve treatment accuracy. Subsequent studies should strengthen the standardization of the experimental design of traditional Chinese medicine clinical research, strictly following the standard guidelines when reporting results. Therefore, clinical research in traditional Chinese medicine could be standardized according to the criteria of evidence-based medicine to realize the guidelines for the prescription of traditional Chinese medicine in a more scientifical and effective manner.

### 4.5. Guidance to clinical practice

Despite these limitations, this NMA provided a reasonable outline and recommendations for the clinical use of CPMs for the treatment of vitiligo. When combined with traditional Western medicine, CPMs may be a good option as long-term oral medication. QT has the best efficacy, but its safety may be worse than that of other drugs, making it suitable for the short-term oral treatment of vitiligo. VCP and QP can be used as first-choice long-term medications. In addition, TP may improve repigmentation in patients with limited lesion areas. Therefore, clinicians should consider the pathogenic site, lesion area, degree of depigmentation, and patient treatment compliance, as well as the theory of traditional Chinese medicine, to choose the appropriate CPM for the long-term oral treatment of vitiligo.

## Acknowledgments

We would like to thank Editage (www.editage.cn) for English language editing.

## Author contributions

**Data curation:** Dingding Wang.

**Methodology:** Jianfeng Wang.

**Writing – original draft:** Jianfeng Wang.

**Writing – review & editing:** Guomin Si.

## Supplementary Material
