# Supplementary material for: Efficacy and safety of Chinese patent medicine compound preparation combined with routine treatment in vitiligo: A Bayesian network meta-analysis
Source: Medicine (Baltimore). 2023 Oct 13;102(41):e35327. doi: 10.1097/MD.0000000000035327 (PMC10578774; doi:10.1097/MD.0000000000035327)
Supplement: Supplementary file 1 [file medi-102-e35327-s001.docx]

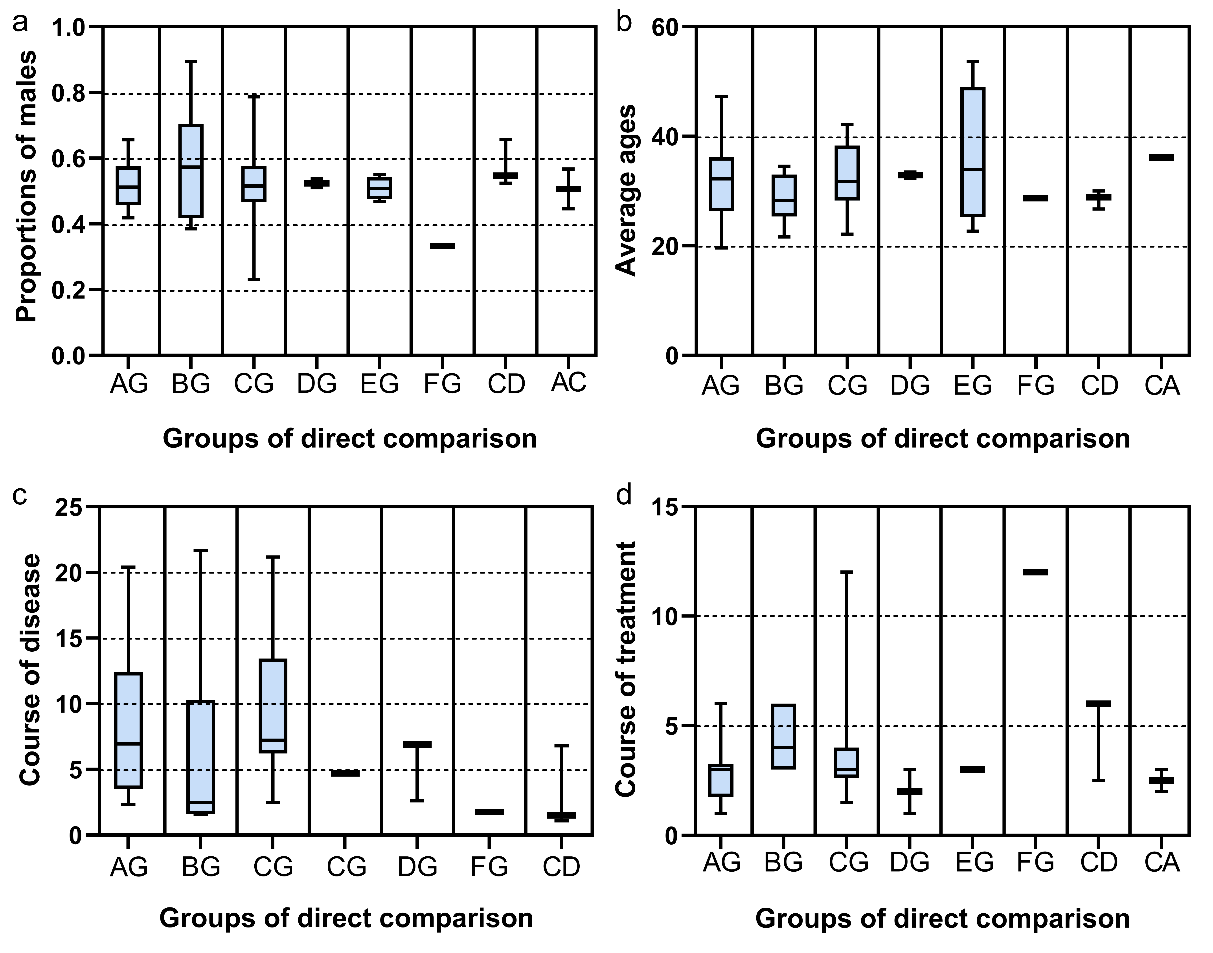


**Supplemental Figure. 1 Transitivity assessment of all the included studies**

(a) Comparison of male participants among the included studies

This figure shows the total evaluation average and 95% confidence interval of the proportion of male patients in each data set directly compared in this study. The short horizontal line in the middle represents the point estimate of the total average, and the two ends of the line segment represent the upper and lower limits of the confidence interval

(b) Comparison of participant age among the included studies

This figure shows the total evaluation average and 95% confidence interval of participant age in each data set directly compared in this study. The short horizontal line in the middle represents the point estimate of the total average, and the two ends of the line segment represent the upper and lower limits of the confidence interval.

(c) Comparison of participant disease course among the included studies

This figure shows the total evaluation average and 95% confidence interval of participant disease course in each data set directly compared in the study. The short horizontal line in the middle represents the point estimate of the total average, and the two ends of the line segment represent the upper and lower limits of the confidence interval.

(d) Comparison of treatment courses among the included studies

This figure shows the total evaluation average and 95% confidence interval of participant treatment course in each data set directly compared in the study. The short horizontal line in the middle represents the point estimate of the total average, and the two ends of the line segment represent the upper and lower limits of the confidence interval.

Abbreviation: A=Vitiligo Capsules or Pills (VCP), B= Bailing Tablets or Capsules (BTC), C= Qubai Babuqi Tablets (QT), D= Baishi Pills (BP), E= Taohong Qingxue Pills (TP), F= compound Quchongbanjiuju Pills (QP), G=Routine Therapy (RT).
