# Supplementary material for: Efficacy and safety of Chinese patent medicine compound preparation combined with routine treatment in vitiligo: A Bayesian network meta-analysis
Source: Medicine (Baltimore). 2023 Oct 13;102(41):e35327. doi: 10.1097/MD.0000000000035327 (PMC10578774; doi:10.1097/MD.0000000000035327)
Supplement: Supplementary file 2 [file medi-102-e35327-s002.docx]

**Supplemental Table 1.** SUCRA values and MeanRank of all treatments

| Outcome Indicators | Treatment | SUCRA (%) | Best Probability (%) | MeanRank |
| --- | --- | --- | --- | --- |
| Total effective rate | VCP | 55.5 | 1.1 | 3.7 |
|  | BTC | 49.0 | 2.7 | 4.1 |
|  | QT | 92.2 | 62.7 | 1.5 |
|  | BP | 35.7 | 0.6 | 4.9 |
|  | TP | 63.8 | 9.5 | 3.2 |
|  | QP | 50.7 | 23.4 | 4.0 |
|  | RT | 3.1 | 0.0 | 6.8 |
| Good improvement rate | VCP | 71.5 | 4.1 | 2.7 |
|  | BTC | 39.7 | 0.5 | 4.6 |
|  | QT | 92.0 | 58.4 | 1.5 |
|  | BP | 36.6 | 0.1 | 4.8 |
|  | TP | 43.0 | 1.2 | 4.4 |
|  | QP | 66.6 | 35.7 | 3.0 |
|  | RT | 0.6 | 0.0 | 7.0 |
| Adverse reactions | VCP | 64.7 | 5.6 | 3.1 |
|  | BTC | 48.0 | 2.8 | 4.1 |
|  | QT | 21.8 | 0.3 | 5.7 |
|  | BP | 42.7 | 22.2 | 4.4 |
|  | TP | 49.2 | 5.3 | 4.1 |
|  | QP | 83.7 | 63.7 | 2.0 |
|  | RT | 39.9 | 0.1 | 4.6 |
| Skin lesion pigment score | VCP | 51.8 | 23.5 | 2.0 |
|  | TP | 83.1 | 72.9 | 1.3 |
|  | RT | 15.1 | 3.6 | 2.7 |
| Lesion area | VCP | 100.0 | 100.0 | 1.0 |
|  | TP | 49.5 | 0.0 | 2.0 |
|  | RT | 0.5 | 0.0 | 3.0 |
| Quality-of-life score | VCP | 63.1 | 29.3 | 2.1 |
|  | QT | 58.5 | 22.5 | 2.2 |
|  | TP | 74.6 | 48.2 | 1.8 |
|  | RT | 3.8 | 0.0 | 3.9 |
